# A Toolbox to Investigate the Impact of Impaired Oxygen Delivery in Experimental Disease Models

**DOI:** 10.3389/fmed.2022.869372

**Published:** 2022-05-16

**Authors:** Stefan Hof, Carsten Marcus, Anne Kuebart, Jan Schulz, Richard Truse, Annika Raupach, Inge Bauer, Ulrich Flögel, Olaf Picker, Anna Herminghaus, Sebastian Temme

**Affiliations:** ^1^Department of Anaesthesiology, Medical Faculty and University Hospital Düsseldorf, Heinrich-Heine-University Düsseldorf, Düsseldorf, Germany; ^2^Experimental Cardiovascular Imaging, Department of Molecular Cardiology, Medical Faculty and University Hospital Düsseldorf, Heinrich-Heine-University Düsseldorf, Düsseldorf, Germany

**Keywords:** microcirculation, sepsis, hemorrhagic shock, mitochondria, MRI, ^19^F MRI

## Abstract

Impaired oxygen utilization is the underlying pathophysiological process in different shock states. Clinically most important are septic and hemorrhagic shock, which comprise more than 75% of all clinical cases of shock. Both forms lead to severe dysfunction of the microcirculation and the mitochondria that can cause or further aggravate tissue damage and inflammation. However, the detailed mechanisms of acute and long-term effects of impaired oxygen utilization are still elusive. Importantly, a defective oxygen exploitation can impact multiple organs simultaneously and organ damage can be aggravated due to intense organ cross-talk or the presence of a systemic inflammatory response. Complexity is further increased through a large heterogeneity in the human population, differences in genetics, age and gender, comorbidities or disease history. To gain a deeper understanding of the principles, mechanisms, interconnections and consequences of impaired oxygen delivery and utilization, interdisciplinary preclinical as well as clinical research is required. In this review, we provide a “tool-box” that covers widely used animal disease models for septic and hemorrhagic shock and methods to determine the structure and function of the microcirculation as well as mitochondrial function. Furthermore, we suggest magnetic resonance imaging as a multimodal imaging platform to noninvasively assess the consequences of impaired oxygen delivery on organ function, cell metabolism, alterations in tissue textures or inflammation. Combining structural and functional analyses of oxygen delivery and utilization in animal models with additional data obtained by multiparametric MRI-based techniques can help to unravel mechanisms underlying immediate effects as well as long-term consequences of impaired oxygen delivery on multiple organs and may narrow the gap between experimental preclinical research and the human patient.

## Introduction

Impaired oxygen delivery and utilization are caused by various diseases with high morbidity and mortality. Many of the mechanisms leading to dysfunctional oxygen exploitation are known, but the acute effects and long-term consequences, as well as their underlying mechanisms, are only poorly understood. Impaired oxygen delivery can impact multiple organs simultaneously, and organ damage can be aggravated by an intense organ cross-talk or the presence of a systemic inflammatory response. In addition to immediate effects, oxygen deprivation might also lead to long-term alterations due to the reprogramming of cells and tissue.

An imbalance between cellular oxygen demand and oxygen supply induces complex adaptive mechanisms in tissues and cells (1, 2). If those mechanisms fail to maintain oxygen delivery and utilization, a shock state establishes. The most frequent forms of shock are acute hemorrhagic and septic shock that are related to high mortality and comprise more than 75% of all clinical cases of shock (3). Importantly, shock-induced impaired oxygen exploitation can occur at multiple sites like the macrocirculation and the local tissue-microcirculation. Of note, local microcirculation does not always correlate with systemic hemodynamics and tissue hypoperfusion can occur despite normal systemic and regional blood flow (4). This indicates the crucial importance to monitor microcirculatory parameters like the structure of the microvasculature, tissue perfusion and oxygen levels or local hemoglobin concentrations. Oxygen utilization is closely connected to mitochondrial function. Mitochondria are the crucial organelles for cellular energy generation, being responsible for about 90% of total oxygen consumption in mammalian cells, 80% of which is coupled to ATP synthesis.

Experimental preclinical animal models in combination with technologies to determine macro- and microvascular structure and function, energy metabolism, tissue damage and subsequent inflammation and organ function can help to gain important novel mechanistic insights into short- and long-term consequences of impaired oxygen delivery and utilization. In this review, we provide an overview about the advantages and limitations of the most widely used animal models for sepsis/septic shock and hemorrhagic shock (Section “Animal Disease Models to Study Hemorrhagic and Septic Shock”) and we describe technologies to measure a broad spectrum of microcirculatory characteristics like the structure of the microvasculature, perfusion, local oxygen levels or mitochondrial function (Section “Technical Devices to Determine Structure and Function of Tissue Microcirculation”). In the Section “Imaging the Consequences of Impaired Oxygen Delivery by MRI” we discuss possible applications of magnetic resonance imaging (MRI) which is a whole-body multimodal imaging technology that can complement and extend the information obtained by techniques described in the Section “Technical Devices to Determine Structure and Function of Tissue Microcirculation”. Moreover, we provide a supplementary overview of at least some preclinical animal studied, where structural or functional aspects of the microcirculation have been determined by imaging technologies (Supplementary Table S1). In the last part of the manuscript (Section “From Bench to Bedside”), we display a brief outlook of how the information obtained by preclinical animal models and technologies to determine microcirculation, mitochondrial function and MRI can be used to obtain complementary information that could be integrated to gain a data-set that might help the clinician for an individual decision making.

## Animal Disease Models to Study Hemorrhagic and Septic Shock

In general, animal models should be characterized by a high grade of reproducibility and a realistic depiction of pathophysiological changes that are also found in the corresponding human shock states. The most common small animal models are based on rodents (5), because they are widely available, experiments with these animals cause relatively low costs and there is a large amount of experimental evidence available that can be used for the design of the study and also for comparison of the results. Mice and to a lesser extent rats, allow for a detailed analysis of the impact of certain genes, or even point mutation for a specific disease state under *in vivo* conditions (6). However, their small size hampers surgical interventions and instrumentation (7), and the small total blood volume prohibits intermittent blood gas analysis from determining base excess (8, 9) and lactate levels (10) as metabolic surrogates for adequate tissue perfusion. Due to their larger size, rats offer the opportunity to perform more laborious surgical interventions, invasive monitoring (11) and also intermittent blood gas analysis. Disease models based on mice and rats allow for a high degree of standardization and a detailed analysis of the pathophysiological mechanism. However, one has to keep in mind that the physiological and immunological response to blood loss or sepsis may differ from humans (12, 13). Shock models in pigs or sheep are more similar to human disease under physiological and hemodynamic aspects and do also enable more clinically relevant surgical interventions. However, the requirements for surgical interventions and for animal care are very high, which renders experiments with these animals rather expensive. They are only available in specialized facilities and therefore will not be covered in this review. Taken together, there is no optimal animal model for modeling hemorrhagic or septic shock which perfectly mimics the human situation. Therefore, the specific research questions, individual experience as well as financial and practical aspects should be carefully considered before planning an animal study.

### Hemorrhagic Shock

The state of hemorrhagic shock is defined as a mismatch of oxygen delivery and cellular oxygen demand due to a loss of cellular and noncellular blood components during critical bleeding (3). Hemorrhage leads to tissue hypoperfusion and to a shift to anaerobic cell metabolism (14, 15). During the last years, the pathophysiological understanding of hemorrhagic shock states developed from a hemodynamic and mechanistic point of view to a more complex process including a differentiated neuronal (16), humoral, (17), microcirculatory and immunological response (18). To gain deeper insights into the mechanisms associated with hemorrhagic shock, multiple small animal models were developed which can be roughly subdivided into (i) controlled hemorrhage, (ii) uncontrolled hemorrhage or (iii) hemorrhagic shock combined with traumatic injury (see below and Table 1 for an overview).

**Table 1:** Experimental models of hemorrhage and their advantages / disadvantages and the specific areas which they are used for.

	**Advantages**	**Disadvantages**	**Utilization**
**Fixed-volume hemorrhage (FVH)**	Reproducible; Standardized; Easy to perform; Linked to ATLS shock-classification	Individual compensatory capacity Differences in shock depth in the late course of shock Differences between the estimated and the actual total blood volume	Investigation of cardiovascular compensatory mechanisms (e.g., adrenergic activation, RAAS)
**Fixed-pressure hemorrhage (FPH)**	Reproducible; Standardized; Easy to perform; Stable shock depth without individual compensation; A clinical and macro-hemodynamic point of view on shock processes	No standardized registration of lost blood volume Focus on hemodynamic changes only	Investigation of hemodynamic coherence and microcirculatory alterations
**Oxygen debt directed hemorrhage (ODH)**	Unique model focusing on oxygen demand as the primary target Reproducible; Standardized	Imprecision in the case of microvascular shunting Not related to tissue-specific oxygen metabolism	To investigate metabolic changes during general hypoxia
**Uncontrolled hemorrhage**	Mimics the clinical situation of uncontrolled and isolated hemorrhage	Limited to a few clinical situations like esophageal- or cancer-bleeding No quantification of total blood volume loss and macrocirculatory hemodynamics High inter-experimental variability	Investigation of physiological cardiovascular changes and internal compensatory mechanisms especially coagulation
**Hemorrhage with traumatic injury**	Close to the clinical situation of severe trauma and surgical bleeding Comprises immunological, metabolic and neurohumoral responses to tissue injury	Very complex and barely comparable due to many different experimental protocols A high number of confounders that impact the results (cytokine release, neuroendocrine activation)	Investigation of immunological, metabolic and neurohumoral changes

#### Controlled Hemorrhage

##### Fixed-Volume Hemorrhage (FVH)

FVH models are characterized by an acute withdrawal of a predetermined blood volume within a short period of time to induce hemorrhage (19). After withdrawal of the defined blood volume, no further interventions, like fluid replacement, are performed. Models differ in the relative amount of the removed final blood volume, the severity of hemorrhage and the duration of hemorrhage. Commonly, the shed blood volume is calculated as the percentage of the estimated total blood volume (20, 21). According to Advanced Trauma Life Support (ATLS), a class IV hemorrhagic shock is apparent when bleeding exceeds 40% of the estimated circulating blood volume which leads to a mortality of more than 30% in humans (22). Therefore, many investigators choose a corresponding shock model to test if a therapeutic intervention results in a survival advantage (23, 24). More moderate shock states are used when the focus is not on survival rates, but, e.g., on repetitive measurements in individual animals (25–27). As stated above, also the shock duration can be adjusted (28). Therefore, investigators should justify shock duration and depth, for example by the individual hypoxia tolerance of the investigated tissues and species. Importantly, the obtained results are not always comparable between species since blood volume is not consistently associated with the total body weight. Of note, even in the same species an increase in body weight does not always result in a linear increase in total blood volume possibly due to a higher percentage of barely perfused adipose tissue (29).

##### Fixed-Pressure Hemorrhage (FPH)

To integrate hemodynamic considerations into the experimental protocol of hemorrhagic shock Wiggers (30) and Penfield (31) were the first to use a fixed-pressure hemorrhage model. In this model, blood is constantly removed until a defined mean arterial blood pressure is reached. To account for compensation or further disarrangements, intermittent bleeding or volume substitution is used to maintain blood pressure.

An FPH model gives the opportunity to investigate microcirculatory alterations independent of macrocirculatory parameters. Especially when changes in the macrocirculation are not paralleled by changes in microcirculatory variables, a FPH model could illuminate new aspects of microcirculatory failure. In this case, the dissociation of micro- and macrocirculatory variables is called hemodynamic incoherence, a field of growing interest in critical care medicine (32). In FPH models, changes in microcirculatory variables after pharmacological interventions may not be reflected in macrocirculatory alterations and probably are a result of adaptive processes within the microcirculation itself. Of note, the FPH model mirrors the clinical point of view, because during the onset of acute hemorrhage physicians first evaluate the macrocirculation to determine hemodynamic stability. Moreover, the total blood loss and especially the relative amount of total circulating blood volume is often difficult to determine in the clinical setting.

##### Oxygen Debt Directed Hemorrhage (ODH)

In the experimental hemorrhage models mentioned above, total blood volume loss or mean arterial pressure is modified, because they impact the maximal blood oxygen content and also tissue perfusion. In ODH, blood loss is controlled by oxygen debt, which is defined as the difference between prehemorrhage oxygen consumption and oxygen consumption during hemorrhage. Oxygen debt is calculated from macrocirculatory variables and blood gas analysis. During hemorrhage, oxygen-carrying capacity decreases due to a reduction of circulating erythrocytes and blood convection within the microcirculation (9). Here it is important to note that systemic oxygen debt often coincides with local tissue hypoperfusion, probably to a varying degree in individual organs, but that these terms are not fully interchangeable.

Oxygen debt is measured for 30 years as a secondary parameter, but Dunham et al. were the first who used this as a target measurement to guide hemorrhage in dogs (33). Furthermore, in follow-up studies they could show, that oxygen debt in hemorrhage is an independent predictor of death and organ failure (34). Crowell and Smith figured out that an oxygen debt of 100 ml/kg or less was not associated with death, whereas an increase of up to 120 ml/kg led to a mortality of 50% (14). Interestingly, this specific correlation seems to be species-dependent (35).

Microcirculatory disarrangements may limit the use of oxygen debt directed hemorrhage models in microvascular research, since blood shunted within the microcirculation is not deoxygenated (36). The resulting increase in postcapillary oxygen content and central venous oxygen saturation may assume a decreased oxygen demand while mortality can be even increased (37). Oxygen debt is calculated from macrocirculatory parameters and represents the systemic oxygen deficit. Those hemorrhage models do not account for the tissue-specific oxygen demand (38). For example, adipose individuals need a lower oxygen amount per total body weight to maintain their basic metabolism (39).

#### Uncontrolled Hemorrhage

In uncontrolled hemorrhage models, animals undergo an open vascular trauma without further bleeding control. This procedure reflects the clinical setting of uncontrolled traumatic bleeding. Thus, the rate of blood loss and hypoperfusion underlies a wide range of variations between individuals and can be barely quantified. Studies using uncontrolled hemorrhage are suitable to investigate internal compensatory mechanisms like coagulation (40). In the past, uncontrolled hemorrhage models were utilized to investigate therapeutical interventions and resuscitation protocols in uncontrolled surgical bleeding (41, 42), that resulted in a paradigm change in fluid resuscitation, pointing toward permissive hypotension in uncontrolled bleeding (43).

#### Hemorrhagic Shock Combined With Traumatic Injury

Isolated hemorrhage barely reflects clinical situations, because hemorrhage usually occurs in combination with tissue injury as a result of surgical interventions or trauma. Tissue injury itself can then cause additional damage through immunological (44) or neurohumoral responses (45). For example, Chaudry et al. observed an increased mortality when laparotomy was performed in addition to hemorrhage (46).

Generally, fixed-pressure models or uncontrolled hemorrhage are combined with additional tissue damage (16). Those models pay attention to neurohumoral, endocrinological and immunological changes during hemorrhage. However, the high complexity of the experimental protocols and the physiological cross-talk of hemorrhage and trauma renders the interpretation and comparison with other studies difficult. Indeed, not only the diversity of possible trauma categories, but different experimental protocols to achieve a certain trauma category are manifold. For example, long bone fractures can be performed as open or close fracture models. Bone fragment implantation and bone marrow injection are called pseudo-fractures and are expected to imitate the immunological effect of long bone fractures without a soft tissue damage through open fracture types (16).

#### Brief Summary of Experimental Hemorrhagic Shock Models

All controlled hemorrhage models enable a high grade of standardization and reproducibility and are therefore suitable for experimental protocols in life science (47) (see Table 1). Of note, all hemorrhage models are defined by the initiation of shock, whereas the posthemorrhagic period underlies a high grade of variation. For example, resuscitation in a FVH model can be achieved by retransfusion of blood, by an increase of mean arterial pressure to a predetermined threshold, or a normalization of base deficit and lactate acidemia through blood, fluid and/or vasopressor substitution and some investigators do not perform resuscitation at all. Reperfusion and resuscitation have a large impact on tissue damage (48, 49), interventions undertaken post hemorrhage have to be reported in detail to allow for a valid interpretation of findings. Importantly, our pathophysiological understanding of hemorrhagic shock states has changed during the last years from a hemodynamic point of view to a more complex process including neuronal, humoral, microcirculatory and immunological aspects. This huge complexity is covered by uncontrolled and combined hemorrhage models that are associated with experimental trauma and tissue injury. However, the large variability within the models makes a standardized application of those shock models in life science challenging.

### Sepsis and Septic Shock

Sepsis is defined as a “life-threatening organ dysfunction caused by a dysregulated host response to infection”. If the severity of these dysregulations is associated with additional hypotension and increased serum lactate >2 mmol/l (18 mg/dl), which substantially increases the mortality, the condition is referred to as septic shock (50). Sepsis remains one of the main problems in healthcare, especially in the ICU (intensive care unit), as sepsis and septic shock are associated with a mortality of about 40% (51). However, the availability of effective supportive therapies that can complement, replace or even prevent treatment with antibiotics and surgical source control are very limited (52). Therefore, basic research is urgently needed to gain a better understanding of the underlying mechanisms that may improve the transfer from preclinical research to clinical usability. In the last century, many different animal sepsis models have been developed that aim to closely mimic human sepsis. In the following paragraph, we provide an overview of the most frequently used sepsis models in rodents.

#### Lipopolysaccharide (LPS) or Bacterial Injection Models

One of the first models was based on an intraperitoneal or intravenous injection of lipopolysaccharide (LPS, also referred to as endotoxin). LPS is a purified part of the wall of gram-negative bacteria (53) and injection of LPS into animals results in similarities to septic shock in humans, like elevated cytokine levels. However, there are fundamental differences in the cytokine dynamics, as cytokine levels rise and fall much faster than in humans (54). This might explain why clinical studies showed no beneficial effect of cytokine inhibiting agents blocking tumor necrosis factor-α or interleukin-1 (55). Many authors consent, that using LPS is not useful for finding an adequate sepsis therapy, as it is absent in gram-positive pathogens, but can be of use to understand the pathophysiology of single aspects of sepsis (53–57).

To more closely mimic human sepsis, but maintaining the easy handling and good reproducibility, different research groups have developed models based on intravenous or intraperitoneal injection of viable bacteria like *Escherichia coli (E. coli), Pseudomonas aeruginosa* or *Bacteroides fragilis* (58–60). Intravenous bolus administration of *E. coli* leads to similar cytokine dynamics like the LPS models. Therefore, injection of bacteria leads to an endotoxin intoxication rather than induction of a true sepsis (61). This is the reason why several authors criticized the bacterial inoculation model and recommended a continuous bacterial infusion to simulate continuous bacterial release as it is present in human abdominal sepsis (61, 62). The combination of different aerobic and anaerobic pathogens covers a broader range of pathomechanisms than the LPS models. In addition to the choice of pathogens, the severity of the induced sepsis can be modified by the amount of colony-forming units and the specific strain (63).

A large proportion of septic patients suffer from respiratory infections, that are the original cause of sepsis (64). To mimic the human situation in experimental disease models, several investigators induce respiratory infection by administration of bacteria either directly into the lungs, or infuse them intratracheally or intranasally (65). These models helped to better understand the pathophysiology of bacterial, but also of viral pneumonia (66). However, investigators should take into account that besides systemic effects of generalized infections, the local tissue damage at the primary source of infection can impact the course of sepsis.

#### Fibrin or Fecal Clot Implantation and Cecal Slurry Injection

In 1980, Ahrenholz and Simmons presented a bacterial inoculum model based on a clot composed of fibrin and viable bacteria. The aim was to generate a slow and continuous bacterial release to gain a more realistic simulation of human sepsis compared to previous models (67). Technically, the clot is implanted into the peritoneal cavity *via* a midline laparotomy and can contain single or multiple strains of bacteria, or alternatively feces (63, 68, 69) and the severity of the disease can be adjusted by the choice and proportion of bacteria. Of note, the cardiovascular response that occurred after implantation of the bacterial fibrin clot showed similarities to human septic shock such as a decrease in peripheral vascular resistance and an increase in stroke volume index (70, 71). One disadvantage of this model is that that it mimics an abscess with peritonitis rather than abdominal sepsis (72).

Starr et al. developed a new protocol for cecal slurry (CS) injection that can be used in neonatal and adult mice (73). The aim was to generate a model that mimics neonatal diseases like necrotizing enterocolitis. To this end, cecal components of adult mice were mixed and resuspended with sterile water to generate the CS. Subsequently, the CS was filtered and mixed with phosphate-buffered saline (PBS) and injected intraperitoneally in mice of different age which resulted in sepsis with a severity that correlated with the dose of the CS. This method is particularly useful, as the small size of the neonatal cecum makes the use of a more complex sepsis model like the cecal ligation and puncture (CLP) or colon ascendence stent peritonitis (CASP) (see below) very difficult (74).

#### Cecal Ligation and Puncture and Colon Ascendens Stent Peritonitis

The surgical sepsis model “cecal ligation and puncture” (CLP) is considered to be the “gold standard” for polymicrobial sepsis (52). A median laparotomy is performed under general anesthesia, subsequently a section of the cecum is ligated on its oral part and then perforated by a needle (most commonly 18–25 Gauge) (75). CLP is characterized by a continuous bacterial release, which results in a more realistic simulation of an abdominal sepsis than bacterial injections. Size and number of the perforations, as well as the size of the ligation and the resulting cecal volume have a direct impact on the severity of sepsis in the CLP model (53). Moreover, this model shows an early hyperdynamic and a late hypodynamic phase, that is also present in human sepsis (76). To verify patency of the lesions, it is essential to slightly compress the cecum to push a small amount of feces through the perforations (75). Importantly, preclinical results of CLP models led to improved diagnostics and therapy. For example, interleukin-6 was found to be useful as a biomarker to assess the probability for sepsis mortality or the improved treatment of septic shock patients with low-dose glucocorticoids (77).

A variant of the CLP model, the so-called colon ascendens stent peritonitis (CASP) technique, was initially described in 1998 by Zantl et al. in mice and later on transferred to rats (78, 79). Similar to CLP, a median laparotomy is performed under general anesthesia. Subsequently, a stent is placed in the ascending colon and fixed with a surgical suture to induce a colon perforation or anastomotic insufficiency, leading to polymicrobial sepsis. As control, sham-operation is performed where stents are sewed to the cecal wall without inducing a perforation. The severity of the sepsis can be adjusted by removing the stents after a certain period of time (CASPi) (78) or by changing the size of the stents, allowing to study mild sepsis as well as septic shock (80). Moreover, removing the stents after 3 h has been used to simulate infectious source control and completely abolished mortality (78). The major difference between CLP and CASP is the resulting disease. CLP shows a more localized inflammation, similar to an intraabdominal abscess, while CASP leads to diffuse, generalized peritonitis mostly seen in patients with anastomotic insufficiency (81).

#### Brief Summary of Experimental Sepsis and Septic Shock Models

The ideal sepsis model should “accurately reproduce the human disease” (82), which implies, that—in terms of abdominal sepsis—it should be polymicrobial, associated with similar pathophysiological changes and with a comparable rate of mortality. The main advantage of the LPS models is that they are highly standardized and easier to perform than surgical procedures like CASP or CLP. However, the most important limitation is that it does not adequately represent the complexity of the human pathology. Therefore, LPS- or bacterial injection models can be used to investigate the relevance of specific questions like the impact of certain cytokine pathways in sepsis (52). Application of pellets that contain bacteria or feces is very suitable to depict peritonitis, but has limited value for abdominal sepsis (57). Utilization of defined bacterial strains strongly reduces complexity, but does not display the polymicrobial character of human sepsis. However, a compromise could be to use defined mixed bacterial cultures (63). Those models that display the highest similarity to human abdominal sepsis like perforated diverticulosis, cholecystitis, bowel ischemia or anastomotic insufficiency are CLP and CASP. In both models, there is a high interindividual variability in the amount of feces that is released into the abdominal cavity (74). However, it should be kept in mind that CASP leads to abdominal sepsis, whereas CLP induces an abdominal abscess. Taken together, every model has its advantages and disadvantages (Table 2), and it should be carefully evaluated which model is suitable to provide the insights that are necessary to answer a specific research question.

**Table 2:** Experimental sepsis models and their specific advantages and disadvantages and the research areas where these models are useful for.

	**Advantages**	**Disadvantages**	**Utilization**
**LPS / Bacterial Inoculation**	Easy handling; No surgical trauma; Dose of LPS or bacteria can be standardized and used to adjust severity of disease	Leads to endotoxemia and septic shock Does not reflect the complexity of polymicrobial human sepsis	Insights into specific mechanisms or pathways associated with sepsis
**Fibrin Clot**	Low mortality and slow septic progress Continuous bacterial release	Surgical trauma Less bacterial variation if used with single bacterial strains	Useful to investigate abdominal peritonitis
**Cecal slurry**	No surgical trauma; Sepsis severity can be adjusted; Polymicrobial	Lethality varies between different donors	Sepsis especially in neonatal mice
**CLP**	Similar to human sepsis; Severity of disease can be adjusted; Polymicrobial	Variability depending on the size of the cecal ligation/perforation	Abscess formation with sepsis
**CASP**	Similar to human sepsis; Sepsis severity can be adjusted; Polymicrobial; Lower variability than CLP	Surgical trauma Time-consuming	Abdominal sepsis Investigation of surgical intervention (CASPi)

## Technical Devices to Determine Structure and Function of Tissue Microcirculation

In the previous section, we described animal models that can be used to investigate certain aspects of hemorrhagic and septic shock. All shock-forms severely affect the local microcirculation that subsequently leads to impaired oxygen delivery and/or utilization. This has profound impact on organ function and can also lead to tissue damage. To gain a better understanding of how shock forms affect the local oxygen exploitation, it is important to investigate how microcirculatory parameters are altered in certain animal disease models. In the following paragraphs, we provide an overview about methods to assess the structure (Section “Evaluating the Structure of the Microcirculation”), and functional aspects of the microcirculation such as perfusion (Section “Microvascular Perfusion: Laser Doppler Flowmetry or Regional Capnometry”), oxygen levels (Section “Oxygen Levels and Hemoglobin”) and mitochondrial function (Section “Mitochondrial Function”).

### Evaluating the Structure of the Microcirculation

The term “structural microcirculation” takes into account that a heterogeneous tissue perfusion can result in hypoxic areas while the total microcirculatory perfusion is maintained (83). Landmark papers from De Backer (84), Ince (85) and Trzeciak (86) found a discrepancy between macro- and microcirculatory variables in septic patients and suspected microcirculatory parameters to be more sensitive to predict multiorgan failure and mortality. This has led to the development of guidelines for the evaluation of microcirculatory alterations in critically ill patients (87, 88). Thus, assessment of microcirculatory status is still a serious clinical challenge, especially under pathological circumstances, like shock. Tissue perfusion is a very dynamic process that can be visualized by videomicroscopic techniques like nailfold videocapillaroscopy or hand-held vital microscopy (HVM).

Nailfold videocapillaroscopy has been used at the bedside to evaluate structural abnormalities in chronic diseases such as vasculitis (89) and diabetes (90). To this end, fingers are placed on the stage of an ordinary microscope, covered with oil and then the capillary density, microvascular blood flow and the averaged vessel thickness can be evaluated and results are interpolated to the general microvascular status. Unfortunately, nailfold videocapillaroscopy is not suitable to evaluate structural microcirculation in shock states due to the high responsiveness of the nailfold arterioles to vasoconstrictor effects of catecholamines, which are standard therapeutics for any kind of shock. Furthermore, this technique can also barely utilized for other parts of the human body.

**Figure 1:**
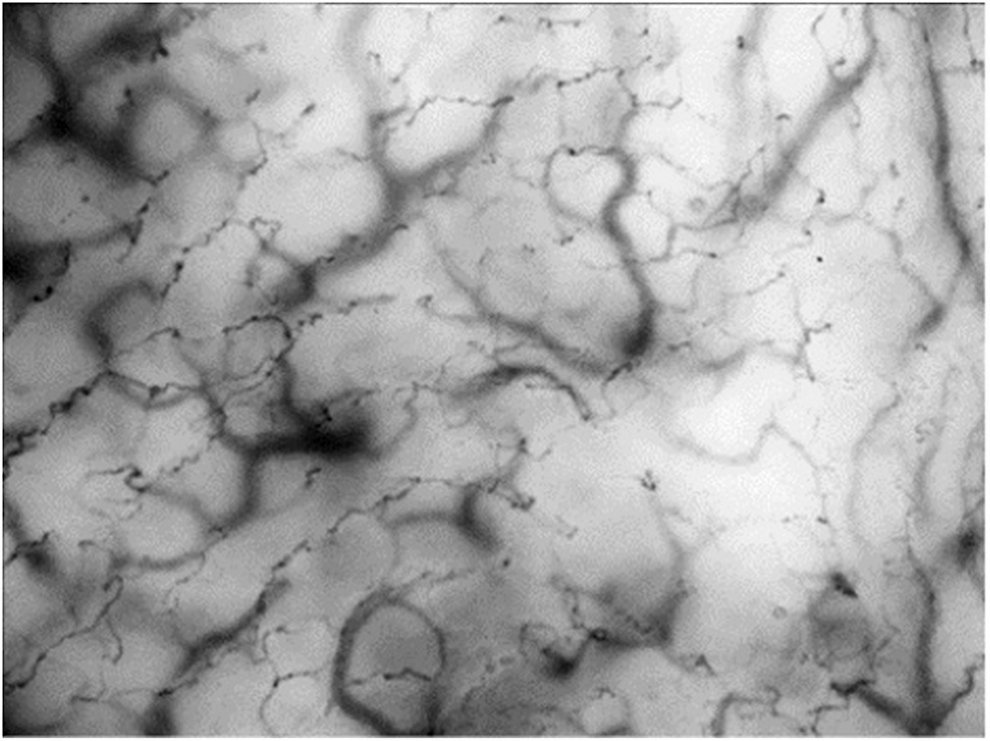
Incident dark-field (IDF-) imaging of oral microcirculation in dogs using hand-held vital microscopy (HVM). Red blood cells are visualized as dark spots forming vessels through their axial migration. According to manufactures IDF-devices are able to register a field of view with 1.55 × 1.16 mm. Oral microcirculation is shown with an additional magnification factor of 4.

HVM-devices were developed to visualize the structural microcirculation and were primarily used for imaging sublingual vessels. First-generation HVM-devices were based on orthogonally polarized spectrum (OPS-) imaging (91), but are no longer available. The second generation utilizes sidestream (SDF-) dark-field imaging. Green-light electrodes surrounding the detector field are emitting pulsed light with a wavelength of 530 nm, which is the isobestic point for hemoglobin. This means that the obtained image is composed of the amount of absorbed light, which is independent of hemoglobin's oxygenation state and the light reflected by deep layers of the investigated tissue. Erythrocytes appear as dark spots and generate the vessel images *via* their axial migration (92). The variable focus of a complex lens system determines the penetration depth and enables the assignment to a specific tissue layer. The third generation HVM is characterized by a very high grade of optical resolution and image quality (for an example image, see Figure 1). The underlying principle is called incident dark-field (IDF-) imaging and it employs a high-resolution imaging chip and short pulsed illumination source that are synchronized to control illumination and image acquisition (93). Aykut et al. performed a prospective, observational study to compare SDF- and IDF-imaging devices and came to the conclusion, that IDF showed increased contrast, sharpness, and image quality for venules and capillaries and therefore gained an improved imaging modality for clinical assessment of microcirculatory alterations (94). Many research groups used HVM to evaluate the microcirculation, particularly in the sublingual area, of critically ill patients.

The main advantage of HVM is the simultaneous evaluation of tissue perfusion and oxygen diffusion distance. To measure tissue perfusion, the main flow characteristics of erythrocytes are scored from 0 (no flow) to 3 (continuous red blood cell flow) and merged to the averaged value “microvascular flow index” (MFI). More important than the absolute velocity of erythrocytes is the predominant type of vascular flow (stopped-flow vs. high velocity capillaries) and flow continuity (pulsatile, intermittent or continuous). Furthermore, spatial heterogeneity in capillary density can also be estimated by HVM. In this context, De Backer et al. introduced a method to evaluate the functional capillary density (FCD) as a marker of oxygen diffusion distance (87). For a quantitative estimation of microcirculatory parameters, they combined total vessel density (TVD) with flow categories (existent, intermittent or absent) to determine the perfused vessel density (PVD) and to calculate the proportion of perfused vessels (PPV). Finally, depiction of flow characteristics in space-time-diagrams can offer further information on the geometrical and functional structure of microvascular beds, flow profiles and the rolling and adhesion of leukocytes (95). Even, if IDF-imaging is a method to visualize the structure of the microcirculation, it can provide multiple additional functional parameters like blood convection, flow characteristics and inflammatory response. In summary, each individual technique has a focus on different aspects of the complex microcirculatory network and the combination of different methods can lead to a more precise picture of the microvascular oxygen delivery and exploitation.

### Microvascular Perfusion: Laser Doppler Flowmetry or Regional Capnometry

Laser doppler flowmetry (LDF) utilizes the doppler shift that is a frequency shift when laser of a specific wavelength is reflected by moving particles or cells (96). The surrounding tissue does also reflect the light, but does not induce a frequency shift and therefore the obtained datasets are composed of shifted and unshifted laser light. LDF provides information about the average blood flow in a variable sample volume that depends on the individual vascularization of the examined tissue, but it is not possible to get information of single microvessels. This means that results obtained with LSF are provided as relative units that pays attention to a variable number of vessels, differences in sampling volume and various directions of blood flow (97).

Adequate tissue perfusion is not only needed for sufficient oxygen delivery, but also crucial for the removal of metabolic products. Thirty years ago, Tang et al. suggested tissue carbon dioxide concentration (p_t_CO_2_) as a marker of adequate tissue perfusion (98). Post resuscitation, an increase in end-expiratory CO_2_ indicates circulatory restoration, which is able to perfuse microvascular beds to wash out carbon dioxide. Moreover, an increasing arteriovenous CO_2_-gap in controlled ventilated patients can indicate a perfusion deficit in high-risk surgery (99). In conclusion, p_t_CO_2_ and especially p_t_CO_2_-p_a_CO_2_-gradients (regional tissue vs arterial CO_2_ partial pressure) may be valuable indicators of tissue perfusion (100). Regional CO_2_-tension can be measured by tonometry or a CO_2_-sensing fluorescence dye (e.g., l-hydroxy-pyrene-3,6,8-trisulfonate (pyranine), shortly HPTS) (101) and can be performed sublingual, endogastral, transcutaneous or in the bladder (102). Technically, saline or air is enclosed by a membrane that is semi-permeable for CO_2_ (103) and after equilibration of the CO_2_-partial pressure between both compartments, the content is aspired and analyzed by infrared spectrometry or conventional gas analysis methods (102).

### Oxygen Levels and Hemoglobin

Oxygen—once released from hemoglobin—follows the gradient of its partial pressure from the vascular space to the intracellular mitochondria. In conclusion, pO_2_-distribution is inhomogeneous within tissues and therefore experimentally determine pO_2_-values are the mean of a local oxygen gradient. The most widely used techniques to determine pO_2_-values are *via* electrodes or phosphorescent probes.

Due to alterations of the structural microcirculation and regional perfusion heterogeneity hemoglobin levels determined from arterial blood gas analysis cannot be transferred directly to the calculation of microvascular variables. Technically, regional hemoglobin levels can be evaluated non-invasively by reflectance spectroscopy (104). In short, white light is emitted into the investigated tissue and scattered at cellular components or absorbed by hemoglobin molecules. The proportion of absorbed light correlates with the total amount of hemoglobin and the difference between emitted and reflected light can be used to calculate the regional hemoglobin concentrations (97). In this case, the hemoglobin content refers to the examined tissue volume instead of the intravascular volume and therefore is named regional hemoglobin concentration (rHb) (104).

Besides the measurement of rHb, light-spectrophotometry can also determine the microvascular hemoglobin oxygen saturation (μHbO_2_). Whereas oxygenated hemoglobin mainly absorbs light with wavelengths about 900 nm at the near infrared spectrum, deoxygenated hemoglobin dominantly absorbs red light with wavelengths about 680 nm. The analysis of the reflected light-spectra allows for calculation of the proportion of oxygenated hemoglobin. Fortunately, this technique is limited to small capillaries since vessels with diameters larger than 100 μm lead to total absorption, due to the high amount of hemoglobin (105). Therefore, light-spectrophotometry is suitable to evaluate microcirculatory variables without any impact of macrocirculatory vessels. Technically, spectrophotometry cannot distinguish between arterial and venous vessels, because the regional volume of the venous system exceeds that of the arteria and therefore μHbO_2_ mainly indicates postcapillary oxygen saturation. Thus, μHbO_2_ does not represent microvascular oxygen delivery, but microvascular oxygen content. However, the correlation of μHbO_2_ and cellular oxygen reserve is only valid if the tissue-perfusion is homogeneous. Shunting within the microcirculation leads to an inhomogeneous tissue-perfusion and an increase in μHbO_2_, which was shown to be related increased mortality (37). Increased μHbO_2_ can falsely suggest a sufficient oxygen delivery, but rather reflects an impaired oxygen utilization due to inhomogeneous microcirculation.

Clark electrodes usually consist of serial platinum wires acting as proton donators. Oxygen diffusion through a semipermeable membrane leads to proton transfer and the formation of OH^−^, which reacts with a corresponding anode (106) and the current is proportional to the oxygen tension (107). Thereby, pO_2_ is measured in a defined volume independent of the grade of vascularization. Results have to be interpreted carefully, because Clark electrodes are typically used in homogeneous samples with a constant oxygen tension. In heterogeneous samples, measurements are more sensitive to the highest values in the sampling volume that leads to an overestimation of the local pO_2_ (108). Measurement of oxygen tension in epithelial cell layers (109) or deeper target tissues can be done with surface- and needle-electrodes that do not affect microcirculatory parameters (110). However, correct placement of the electrodes leads to tissue trauma, which may influence regional metabolism and thus oxygen balance (104).

Palladium-containing porphyrine compounds show an oxygen tension dependent phosphorescence quenching after a pulsed light stimulation (111). To address the fact, that oxygen diffusion through tissues mainly depends on the magnitude of intravascular oxygen tension, palladium porphyrine is conjugated to human albumin to verify that the probe is maintained within the vascular system and does not diffuse into tissue (104). Therefore, palladium porphyrine based methods focus on intravascular oxygen tension. Limitations of this method are the time-dependent decrease in palladium porphyrine concentration and its potential to provoke allergic reactions. Furthermore, palladium-dyes are toxic for humans, which prohibits the use in patients (112).

### Mitochondrial Function

Regarding a very complex cascade of impaired processes by oxygen delivery and utilization in a shock state, it is pivotal to assess also the final step of oxygen consumption taking place in the respiratory chain in the inner mitochondrial membrane. Unfortunately, there are no clinically established methods for bedside measurement of all aspects of the mitochondrial respiration. The assessment of the functionality and efficacy of the respiratory chain can only be performed *in vitro* or *ex vivo* as described in the following chapter. A short insight into the novel but very promising devices for mapping the mitochondrial oxygen consumption in a clinical setting can be found in the last chapter “From bench to bedside” (see section From Bench to Bedside).

#### States of Mitochondrial Respiration

Mitochondria are those intracellular organelles that are responsible for about 90% of total oxygen consumption in mammalian cells, 80% of which is coupled to ATP synthesis. Present in nearly all types of human cells, mitochondria are vital to our survival. Besides their main task the energy production, they are also involved in many other cellular processes, including calcium homeostasis, and regulation of cell death (113).

**Figure 2:**
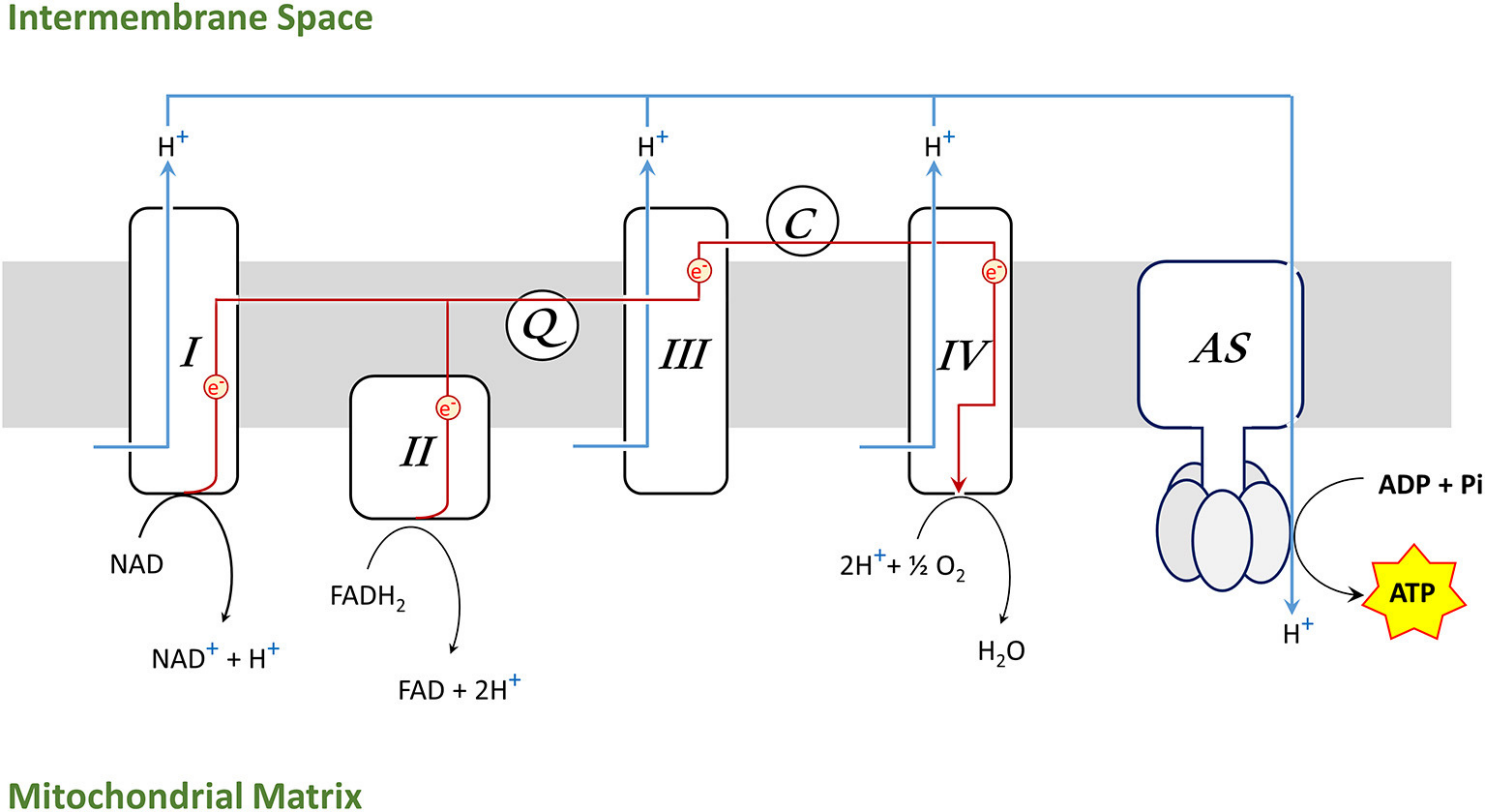
Electron transport chain (ETS) in the inner mitochondrial membrane [complexes I–IV, ATP synthase (AS), coenzyme Q (Q), cytochrome C (C)].

Mitochondrial respiration takes places in the inner mitochondrial membrane through four large protein complexes (I–IV) as well as the adenosine triphosphate (ATP) synthase (AS) (also called complex V). Coenzyme Q (Q) and cytochrome C (C) are diffusible electron carriers between complex II and III (Q) and between complex III and IV (C), respectively (Figure 2).

Complex I oxidizes NADH to NAD^+^ and complex II reduces FADH_2_ to FAD, donating electrons, which are transported along the complexes to generate H_2_O in a reaction involving molecular oxygen and hydrogen protons (H^+^) at complex IV. Simultaneously, complex I, III, and IV are pumping H^+^ across the inner membrane, creating a gradient which drives hydrogen back through AS and provides energy for phosphorylation of adenosine diphosphate (ADP) into ATP. The synthesis of ATP by mitochondria is called oxidative phosphorylation (OXPHOS).

**Figure 3:**
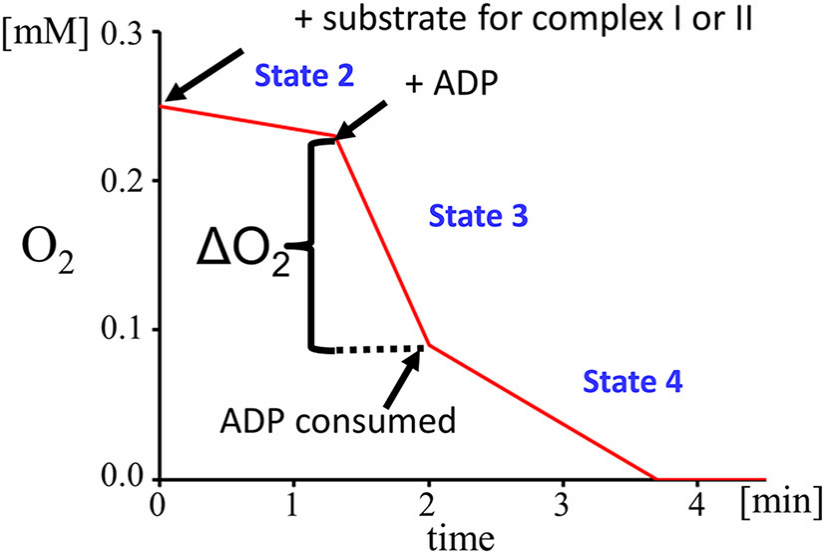
Respiratory states: state 2 (substrate dependent respiration), state 3 (ADP dependent respiration), state 4 (steady state respiration after ADP depletion).

The mechanism that underlies the energy-generating capacity of mitochondria was described by Mitchell in 1961 (114) and awarded with the 1978 Nobel Prize in chemistry. He described four states of mitochondrial respiration, which occur under physiological circumstances and can be simulated by the selective use of different stimulators and inhibitors to gain a detailed insight into the mitochondrial respiration (Figure 3).

Mitochondrial state 1 respiration is a basal form of oxygen consumption that occurs in an environment which is poor in substrates for the electron transport chain and ADP. Mitochondrial state 2 respiration represents a situation with an abundance of substrates for the electron transport chain. Complex I is triggered by glutamate and malate, which both provide NADH as substrate for complex I. Glutamate is oxidized by the enzyme glutamate dehydrogenase to α-ketoglutarate and the malate dehydrogenase oxidizes malate to oxaloacetate. Both reactions are driven by the reduction of NAD^+^ to NADH. In parallel, complex II is fueled by electrons derived from FADH_2_ which is generated *via* the succinate dehydrogenase that converts succinate to fumarate.

To differentiate between the function of complex I and II, complex I can be blocked by adding rotenone prior to the addition of succinate. Rotenone prevents the back-flow of electrons from complex II to complex I. The low level of mitochondrial respiration (state 2) can be altered experimentally by the addition of ADP, which initiates a flow of protons from the intermembrane space to the mitochondrial matrix. This proton gradient provides the energy that is required by the ATP synthase to phosphorylate the added ADP into ATP. This is the so-called state 3 mitochondrial respiration. A limiting factor for state 3 respiration is the concentration of ADP. To define a measure of the coupling between the electron transport chain and the OXPHOS the respiratory control index (RCI) is calculated by dividing state 3 by state 2. Another parameter reflecting the efficiency of OXPHOS is the ADP/O ratio that is calculated from the amount of ADP added and the O_2_-consumption measured during state 3. When ADP is consumed, a steady state of respiration is reached which is called state 4 respiration.

It should be noted that, even though there is only one outcome parameter (oxygen consumption), the conditions of the experiment can be chosen very specifically to link effects to a particular part of the respiratory chain or to a specific enzymatic reaction. Using combinations of different mitochondrial substrates, inhibitors, and stimulating agents, mitochondrial capacity can be characterized extensively and very precisely, just by measuring oxygen consumption under these experimental conditions.

#### Measurement of Mitochondrial Respiration

Measurement of mitochondrial respiration can be performed in either isolated mitochondria, or tissue- and cell-preparations, in which the barrier function of the plasma membrane is either mechanically disrupted or dissolved by application of mild detergents.

Assessment of mitochondrial respiration on isolated mitochondria obtained by differential centrifugation was considered as the gold standard for decades. But there are severe limitations and drawbacks of this isolation technique, as it is impossible to obtain the whole population of the tissue mitochondria. For example, only 40% of cerebral and about 10% of hepatic mitochondria can be retrieved by this procedure (115). Furthermore, the mitochondrial fraction obtained by this isolation technique is not pure but is contaminated with microsomes. Further purification steps are laborious and require time, money, and resources.

As an alternative, tissue homogenates are also routinely used for assessment of mitochondrial oxygen consumption, enzyme activities (116), membrane potential, or protein content (117). The main advantages of using tissue homogenates are: limited workload, stability of the tissue samples, small tissue sample required for measurement (a fivefold of tissue is needed for analogous measurement with isolated mitochondria) (115). This method has also drawbacks like the heterogeneity of the cells (especially in tissues consisting of different cell types like gut or lung) and a large amount of biological active substances such as enzymes, substrates or inhibitors of the respiratory chain which can possibly affect the results. The measured parameters of mitochondrial functions are similar in both tissue homogenates and isolated mitochondria and they are both widely used dependent on research questions, tissue, and study design. It is also possible to measure mitochondrial respiration in cell culture. Of note, assessment of mitochondrial respiration is hampered by the cellular plasma membrane. The plasma membrane consists of a lipid bilayer, proteins, and further organic molecules that prevent the passage of many water-soluble mitochondrial substrates, such as succinate or ADP, that are required for the analysis of mitochondrial respiration. To overcome this difficulty, mild detergents such as digitonin and saponin can be applied to selectively permeabilize the plasma membrane but not the mitochondrial membranes, since the cholesterol content of the plasma membrane is higher compared to mitochondrial membranes.

Different devices based either on polarographic principle (Clark electrode) or on fluorophore's technique are available for the assessment of mitochondrial oxygen consumption. Both methods are suitable for isolated mitochondria, tissue homogenates or cells (see below).

#### Available Devices for Respirometry

##### Polarographic Respirometry for Measurement of Oxygen Consumptions in Isolated Mitochondria, Cells or Tissue Homogenates in a Closed Chamber

The main principle of polarographic respirometry is that oxygen diffuses through a teflon/polypropylene membrane, which is permeable to uncharged gases but not to water. A platinum/silver/KCl coupled electrode reduces oxygen and oxidizes silver, producing a current which is proportional to the oxygen concentration (118). This method can be used for measurements in intact cells, tissue homogenates and isolated mitochondria.

##### Optical Sensor Probes to Determine O_2_ Consumption in Multiwell Plates

Oxygen consumption can also be determined using an optical probe. Changes in oxygen concentration and pH are detected by two fluorophores and automatically calculated and reported as Oxygen Consumption Rate (OCR) and ExtraCellular Acidification Rate (ECAR). The measurements are performed in real time, which enables collecting kinetic data of cell metabolism.

The probes are inserted into the culture medium within a small chamber and placed in close vicinity to the cells. Another possibility to monitor oxygen consumption is using plates coated with porphyrine. Porphyrine molecules are characterized by fluorescence signals in the near-infrared range that are quenched in the presence of oxygen. This technology is mainly used for cytotoxicity measurement, because O_2_ consumption is one of the most sensitive parameters for cell toxicity (119).

## Imaging the Consequences of Impaired Oxygen Delivery by MRI

In the previous chapters, we describe technologies that allow for measurement of structural and functional microcirculation, oxygen levels and mitochondrial activity—processes that are strongly impaired in the acute phase of septic and hermorrhagic shock. These technologies allow for a detailed characterization of the structure and function of the local microcirculation, but they are invasive or only applicable to the surface of specific tissues. Here we present magnetic resonance imaging as a versatile imaging technology that is not limited by penetration depth and which can be utilized for animal models described in section “Animal Disease Models to Study Hemorrhagic and Septic Shock” and can complement the technologies described in the third part of this review. Sepsis and hemorrhagic shock are very complex diseases and MRI as multimodal whole-body imaging technology can acquire parallel information about anatomy, organ function, metabolism, inflammation and thrombotic processes. In the clinical context, MRI is not the optimal technology for the acute phase of critically ill patients, but it can be used to monitor the short- and long-term effects and consequences of impaired oxygen delivery over time in multiple inner organs.

### ^1^H MRI to Determine Anatomy and Function of Organs

The most widely used biomedical application of magnetic resonance imaging is the visualization of the anatomy of inner organs that can be used to detect tumors, abnormalities in the brain tissue or the spinal cord, or to diagnose cardiovascular diseases such as atherosclerotic plaques, the formation of aneurysms or heart disease. MRI can acquire three-dimensional datasets, therefore it does not only determine regional alterations in cross-section images, but can also provide volumetric data of whole organs.

In addition, it is possible to derive multiple types of functional information. Cardiovascular MRI of the heart is used to determine endsystolic-, enddiastolic volumes, stroke volume, ejection fraction and multiple other parameters (120). Brain activity is associated with changes in blood flow and the amount of oxy- and desoxyhemoglobin and these parameters are used by functional MRI (fMRI) to gain information about brain activity (121). One crucial aspect of proper organ functionality is the integrity of the vascular system. MR-angiography (MRA) enables the visualization of the flowing blood, that is used to assess vessel geometry or to calculate pulse-wave velocities to identify stenosis or aneurysms (122). More recently, sophisticated four-dimensional angiographic imaging techniques have been developed that enable a quantitative assessment of time-resolved flow profiles of larger vessels (123). Due to limitations in sensitivity, MRI of small capillaries (microcirculation) is more difficult. To gain information about the functionality of the microcirculatory system, perfusion-imaging techniques have been developed, which are conducted by the application of gadolinium-based contrast agents, the utilization of oxy- and deoxyhemoglobin or arterial spin labeling (124, 125).

Another interesting feature of MRI is that it has the capability to quantitatively characterize alterations in tissue properties. Parametric MR-mapping techniques to determine T1- and T2-relaxation values (T1-, T2-mapping), proton density or CEST (chemical exchange saturation transfer) effects. These mapping techniques provide a quantitative pixel-by-pixel representation of the obtained values for a certain organ (126). T1- or T2-mapping has been used to gain information about the presence of tissue edema or fibrosis (127–129), whereas proton density mapping has been utilized to determine tissue water content (130), hepatic fat (131) or for tissue-texture analysis (132). An important feature of the tissue-texture is the extracellular matrix that contains numerous exchangeable protons—in particular in the hydroxylated sugar moieties of hyaluronic acid and glycosaminoglycans—that can be visualized by CEST (133). CEST-mapping has for example been performed in mice after myocardial infarction or in graves orbitopathy to monitor remodeling of the extracellular matrix (127, 134).

### Magnetic Resonance Spectroscopic Imaging and MRI of X-Nuclei

It is important to note that the magnetic properties of ^1^H-protons are not all identical, but the resonance frequency depends on the chemical structure of the molecule. For example, the ^1^H-protons of water and fat show a chemical shift of 3.4 ppm and therefore, fat and water can be measured separately which has been used to determine alterations in the fat content (135). However, other hydrogen atoms in a certain chemical environment possess distinct chemical shift properties that can be identified *in vivo* by single voxel magnetic resonance spectroscopy (MRS) or multi-voxel MR-spectroscopic imaging [also called chemical shift imaging (CSI)]. These techniques allow for a non-invasive detection and quantification of a number of metabolites from localized volumes within living organisms (136). This has been used to detect N-acetyl aspartate acid, choline, creatine, lactate, myo-inositol and glutamate as well as glutamine in the brain (136). Of note, chemical shift imaging is not limited to ^1^H, but can also be applied to other MR-active nuclei like ^13^C, ^31^P that have been utilized in the past for visualization of metabolites in the heart or the liver (137, 138).

Apart from ^1^H-protons, MRI can detect a number of other nuclei with a nonzero spin quantum number like ^35^Cl, ^31^P, ^39^K, ^17^O, ^13^C, ^23^Na or ^19^F (139, 140) (see Table 3). Detection of these X-nuclei is not used for anatomical purposes, but can provide insights into physiological processes (139, 141). Na^+^, K^+^ and Cl^−^ regulate cell membrane potential and impact the cell volume, whereas ^17^O can provide insights into oxygen consumption. ATP and phosphocreatine are important products of the cellular energy metabolism that can be detected by ^31^P MRI and has been used to get insights into the metabolism of murine hearts (137). Organic molecules contain a backbone of carbon atoms and ^13^C-spectroscopy has been widely used to track organic metabolites (139). However, most of the X-nuclei display a quite low sensitivity compared to the ^1^H nucleus, which makes the detection and imaging quite challenging. One interesting exception is fluorine 19 (^19^F) that has a gyromagnetic ratio close to ^1^H, a natural abundance of 100% and is nearly absent from biological tissues. As a consequence, detection of ^19^F tracer molecules by ^19^F MRI within living organisms can be conducted with high sensitivity and specific and makes this very attractive for a variety of MRI approaches (Section “Tracking of Immune Cell Trafficking by ^1^H/^19^F MRI”).

**Table 3:** NMR-relevant properties of some nuclei. Data is adapted from references (139, 142, 143).

**Element**	**Isotope**	**Nuclear spin**	**Gyromagnetic Ratio (γ) [10^**7**^ × rad × s^**−1**^ × T^**−1**^]**	**Natural abundance [%]**	**NMR frequency (MHz) at 9.4T**
**Hydrogen**	^**1**^ **H**	1/2	26.75	99.98	400,2
**Carbon**	^**13**^ **C**	1/2	6.73	1.07	100.7
**Oxygen**	^**17**^ **O**	5/2	−3.63	0.04	54.3
**Sodium**	^**23**^ **Na**	3/2	7.08	100.00	105.9
**Phosphorous**	^**31**^ **P**	1/2	10.84	100.00	162.2
**Chlorine**	^**35**^ **Cl**	3/2	2.62	75.77	39.3
**Potassium**	^**39**^ **K**	3/2	1.25	93.10	18.7
**Rubidium**	^**87**^ **Rb**	3/2	8.79	27.83	131.4
**Fluorine**	^**19**^ **F**	1/2	25.16	100.00	376.7

Septic and hemorrhagic shock states both coincide with alterations in tissue metabolisms partly due to limited oxygen supply or the action of proinflammatory cytokines. Noninvasive MR-imaging of metabolites like lactate, or the assessment of energy levels (ATP or PCr) could help to identify severely affected organs and to localize and characterize those regions within organs that are suffering from impaired oxygen delivery and exploitation.

### Imaging of Inflammatory Processes by MRI

Both hemorrhagic as well as septic shock are associated with inflammatory processes that contribute to tissue injury and in severe cases can lead to multi organ failure (144, 145). Noninvasive imaging of the associated inflammatory reaction by MRI can help to assess the overall magnitude of the systemic inflammatory response, can identify affected organs or help to unravel novel mechanisms. Direct visualization of inflammatory processes or inflammatory cells by classical MRI is difficult, because particularly in the early phase the affected tissue with infiltrated cells does not cause physical alterations that can be converted into MR-contrast. To overcome this limitation, contrast agents or tracers have been developed that highlight certain aspects of the inflammatory reaction (146).

One of the most important hallmarks of an immune response is the infiltration and accumulation of different kinds of inflammatory cells like neutrophils, monocytes and macrophages or T-cells. One of the most popular MR contrast agents that are used for the visualization of monocytes and macrophages are SPIOs or USPIOs (small/ultra-small paramagnetic iron oxide nanoparticles). SPIOs have a size of 50–100 nm, are strongly internalized by monocytes and macrophages and therefore utilized for detection of tumors (liver) and inflammatory processes (147, 148). USPIOs have a diameter of <50 nm, possess a longer blood retention time and can also be used as contrast agents for MR angiography (149). The major advantage of this approach is a very high sensitivity, but there are also drawbacks. The local accumulation of iron oxide particles leads to the disruption of the ^1^H signal (negative contrast) in this area that can make the identification of correct anatomical localization difficult and there is no linear correlation between signal decay and iron oxide concentration that complicates absolute quantification.

#### Tracking of Immune Cell Trafficking by ^1^H/^19^F MRI

**Figure 4:**
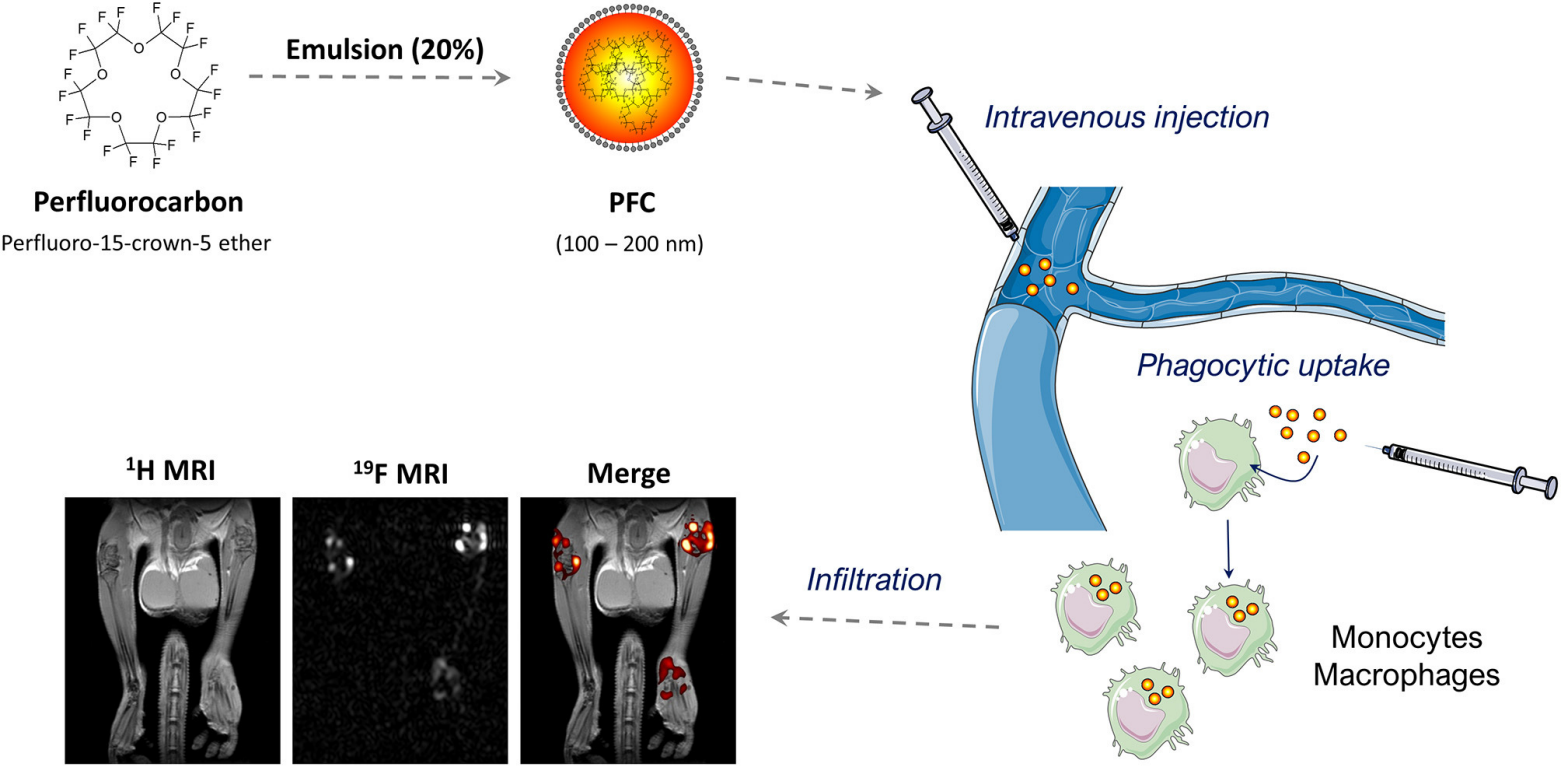
^19^F MRI-based inflammation imaging: Perfluorocarbons are emulsified by high-pressure homogenization to generate perfluorocarbon nanoemulsions (PFCs) with a size between 100 and 200 nm. Intravenous injection of PFCs leads to cellular uptake by circulating or local phagocytic immune cells—particularly monocytes and macrophages. The accumulation of these cells is detected by combined ^1^H/^19^F MRI. The MRI images show the anatomy of legs and hips of a male mouse with collagen-induced arthritis (left), the corresponding ^19^F dataset (middle) and a merging of both (right). Note that the merged image reveals the anatomical localization of the phagocytic immune cells. ^1^H/^19^F MRI images were adapted from Flögel et al. (156), and elements of the schematic figures were derived from: https://smart.servier.com/.

An alternative approach to visualize inflammatory immune cells is based on perfluorocarbon nanoemulsions (PFCs) or nanoparticles that are visualized by ^19^F MRI. The accumulation of ^19^F atoms within organisms can be detected with high sensitivity and specificity and as opposed to iron oxide nanoparticles generates a true positive contrast that does not interfere with the anatomical ^1^H images. Perfluorocarbons are fluorinated organic molecules with a high ^19^F-content that have to be emulsified or encapsulated for biomedical applications (150). High pressure homogenization or microfluidization with lipids as emulsifier leads to the generation of PFC-droplets with a hydrodynamic diameter of 100–200 nm (Figure 4) (150). Alternative methods to generate biocompatible perflurorocarbon ^19^F-tracers are the emulsification with poloxamers or encapsulation into PLGA (Polylactid-co-Glycolid) nanoparticles (151, 152).

Accumulation of PFCs in inflammatory lesions has already been observed in the 1980s (153), but the first systematic investigation that verified the internalization of intravenously administered PFCs by monocytes and macrophages after myocardial infarction and stroke has been published in 2008 (154). Density separation of the blood, flow cytometry and histochemistry verified that PFCs can be internalized by monocytes and that PFCs colocalized with monocytes and macrophages within the infarct area. These findings were confirmed in follow-up studies of this group (155–159) and independent studies from other groups (160–167), which has manifested the concept that intravenously injected PFCs are internalized by phagocytic immune cells that accumulate at inflammatory lesions and can be detected by combined ^1^H/^19^F MRI (Figure 4). However, cellular uptake of PFCs is not purely specific for monocytes/macrophages and under certain circumstances other cells like neutrophils (156, 168) or even specific progenitor cells within the heart (169) can contribute to the local ^19^F signal.

An alternative strategy to visualize immune cell trafficking is the *ex vivo* labeling of cells with PFCs followed by reimplantation and subsequent ^1^H/^19^F MR imaging. This approach is of particular interest for specific cell types that cannot be selectively labeled by intravenous injection of PFCs or for cell-therapeutic approaches like dendritic cells (DC) or T-cells. A pioneering study by Ahrens et al. (170) utilized PFCs to label dendritic cells *ex vivo* and monitored DC migration into the popliteal lymph nodes by ^19^F MRI. In principle, *ex vivo* labeling requires strong phagocytic properties which are absent in T-cells. However, the addition of transfection reagents or the development of specialized PFCs enabled an efficient labeling of T-cells that made it possible to track their migration into lymph nodes or the pancreas (171, 172). *Ex vivo* labeling is an interesting strategy to load isolated cell types, but the isolation and incubation can change the functionality of the cell types and the cell population has to be purified from PFCs that were not internalized during the loading process.

#### Visualization of Thromboinflammatory Processes by Multiplex ^1^H/^19^F MRI

The chemical shift range for ^19^F atoms spans over 400 ppm (173) and some of the perfluorocarbons display individual spectral signatures. Utilizing PFCs with distinct spectral properties enables the detection of several target structures or cell types. The first report for such a multispectral ^19^F MRI was published in 2007 and showed the labeling of human progenitor cells with PFOB- or PFCE-PFCs (174). With raising interest in ^19^F MRI, several recent publications addressed multispectral or multicolor ^19^F MRI for *in vivo* imaging of distinct ^19^F-nanotracers such as nanoemulsions, nanoparticles or even nanocrystals (175–179). These approaches nicely show the feasibility of multispectral ^19^F MRI, but do not combine multispectral ^19^F-MRI with the targeting and visualization of several structures *in vivo*.

Over the past years, our group has established a platform for the active targeting of PFCs and we were able to functionalize PFCs with specific targeting ligands and to use this technology to visualize thrombi (180–182), activated platelets (183), cells expressing synthetic receptors (184) or specific progenitor cells of the heart (185). Moreover, we recently implemented a ^19^F-imaging technology, which enables the simultaneous detection of PFCs with distinct ^19^F spectra and combined this with the simultaneous visualization of factor XIIIa-activity, fibrin and monocytes/macrophages (186). We exposed mice that express a hypomorphic mutant form of apolipoprotein E and also lack the scavenger receptor class B type I to a high fat/high cholesterol diet that leads to a thromboinflammatory phenotype with plaque ruptures and subsequent myocardial infarction (187). Using this multitargeted approach, it was possible to identify high-risk areas at very early time points and importantly the ^19^F signal was predictive for consecutive development of myocardial infarction (186).

### Preclinical MRI of Hemorrhagic and Septic Shock

Although shock states are of high clinical relevance and MRI systems provide a versatile multimodal whole body platform, the number of preclinical studies that utilize MRI to analyze the short- and long term-effects and consequences of hemorrhagic and septic shock is relatively limited. Animal studies have utilized classical ^1^H-based anatomical, functional or parametric MRI mapping techniques to investigate alterations in tissue structure or perfusion in different organs. Below, the reader finds some examples for research studies including MRI measurements that have been conducted in different models of septic and hemorrhagic shock.

#### Septic Shock

Cerebral edema is a frequent finding in sepsis and is caused by breakdown of the blood brain barrier and by energy depletion of neutrons due to microcirculatory failure. Rosengarten et al. addressed the question whether early microcirculatory failure leads to brain edema (188). To this end, the authors utilized a rat model of LPS-induced endotoxemic shock, followed by sequential MRI investigations over several hours. Brain edema was monitored by diffusion weighted MRI and T2-mapping in several regions of the brain. However, the authors did not find alterations in diffusion coefficient or T2-values indicating that at least in this system, microcirculatory failure is not associated with the occurrence of brain edema (188).

Another organ-system that is severely affected by septic shock are the kidneys (189). To gain information about kidney function in polymicrobial cecal ligation and puncture (CLP), a gadolinium-based G4 dendrimer was administered and the accumulation of this agent was observed in the kidneys (190). The authors found that this technique can distinguish between different forms of renal failure but more importantly, that it has the capability to detect renal dysfunction earlier than an increase in serum creatinine. An effect of septic shock on kidney function has also been found by Weidensteiner et al. (191) using dynamic contrast enhanced MRI after CLP. Gadolinium-DTPA (DTPA = diethylenetriaminepentacetate) was used to monitor the accumulation of the contrast agent in the kidneys and in the bladder. Interestingly, CLP led to a delayed washout of the contrast agent from the kidneys into the bladder that indicates impaired glomerular filtration.

It is known that shock can lead to the generation of reactive oxygen species, which can aggravate tissue damage. Visualizing reactive oxygen species by MRI is difficult, because of their very short half-life. To overcome this limitation, spin-trapping has been developed which is a technique that can be used to scavenge reactive oxygen species followed by visualization of the reaction products. Towner et al. investigated the possibility to visualize shock-induced free radicals by combined immuno-spin trapping and molecular MRI after CLP (192). The authors used DMPO (5,5-dimethyl pyrroline N-oxide) to trap reactive oxygen species 6 h after CLP and then administered a gadolinium-labeled anti-DMPO probe for MRI-detection. The authors found increased amounts of DMPO-adducts and elevated oxidative products in septic brains compared to sham controls. Another free radical that is produced by the vascular system under inflammatory conditions is nitrogen oxide (NO). To investigate the generation of NO during LPS-induced sepsis in mice and rats, Fuji et al. also utilized an iron-dithiocarbamate complex ((MGD)_2_-Fe(II) = N-methyl-D-glucamine dithiocarbamate) which leads to the generation of (MGD)_2_-Fe(II)-NO. This reaction product strongly decreases T1- and T2-relaxation of the surrounding water which was exploited to visualize the generation of NO under *in vivo* conditions in the liver tissue and vasculature of rats and mice under septic shock (193).

#### Hemorrhagic Shock

The liver is an important organ that responds to low flow states that are associated with trauma and hemorrhage. Experimental studies have demonstrated that these conditions lead to adhesion of leukocytes to the liver endothelium (194), changes in hepatic macrophages (195) and tissue damage (196). In this context, Matot et al. (197) utilized MRI of the liver in a rat model of hemorrhagic shock to evaluate tissue injury caused by reduced perfusion. Acute hemorrhage led to significantly reduced relative MRI signal changes, reflecting lower liver perfusion. Signal intensity also correlated with the percentage of blood loss. In another experimental study in rats, Maier et al. (198) investigated the impact of hemorrhagic shock on liver function and the activation of hepatic macrophages by MRI. The authors used a liver-specific contrast agent (Gd-EOB-DTPA) that is absorbed by hepatocytes and secreted into the bile fluid as well as SPIOs to label liver macrophages. Application of Gd-EOB-DTPA revealed a reduced secretory function of hepatocytes 24 h after shock, no difference was found after administration of SPIOs. However, the authors observed an activation of liver macrophages by fluorescently labeled latex beads, which could suggest that this MRI-approach is not sensitive enough to visualize the activation of liver macrophages under these conditions.

## From Bench to Bedside

Shock states are commonly characterized by regional hypoxia, anaerobic metabolism and cell damage. Consequently, the optimization of microcirculatory oxygen delivery as well as the maintenance of physiological cell metabolism should be the therapeutic goal to obtain tissue integrity and avoid cell damage. Nowadays, physicians still rely on surrogate markers to guide therapeutic interventions in critical care medicine. Integrating information about novel mechanisms, microcirculation, mitochondrial function and tissue damage with subsequent inflammatory reaction could strongly improve individual therapy decisions.

Patients under severe shock states are barely compensated and suffer from additional comorbidities and confounders that make the situation even more complex. Unfortunately, only a few microvascular beds are accessible that can be used to assess microcirculatory structure and function like the sublingual surface. HVM of sublingual vessels can provide a visual impression of microcirculatory alteration in shock states, but the application of HVM to other sides of the gastrointestinal tract is challenging and restricted to special situations like ICU patients with enterostoma (199) or open abdominal surgery (200, 201). However, imaging of the microcirculation can not only be used for diagnosis but also to monitor therapeutic approaches like the application of vasoactive substances to optimize blood flow kinetics and to prevent total vessel occlusion by pharmacological vasoconstriction (202, 203). Septic patients often receive excessive fluid substitution to maintain normal blood pressure which does not necessarily lead to increased cellular oxygen delivery (204), but results in tissue edema that enlarges the oxygen diffusion distance and may even impair tissue oxygenation.

Monitoring mitochondrial function in real-time *in vivo* is still not employed in clinical settings, but could complement the information obtained from microcirculation measurements, because it provides insights into O_2_ utilization and energy production. One possibility to evaluate the activity of the respiratory chain *in vivo* is to measure the mitochondrial NADH redox state by monitoring UV absorbance or blue fluorescence of NADH (205). This technology still has some limitation like a need for better correction for hemodynamic artifacts and for quantification of the signals (205). Measurement of mitochondrial function in skeletal muscle is based on the recovery of muscle homeostasis after exercise by near-infrared spectroscopy (NIRS) and determines the return of muscle oxygen consumption to basal levels (206). Based on the protoporphyrine IX-triplet state lifetime technique (PpIX-TSLT) (207), Neu et al. established a new measurement protocol for the “Cellular Oxygen Metabolism” (COMET) allowing assessment of mitochondrial oxygen tension, consumption and delivery (208). The first results with septic patients were promising, but further studies in greater cohorts are required to establish potential diagnostic and therapeutic benefit of this method.

One of the disadvantages of the methods described above is that these techniques are mainly limited to superficial tissues. In contrast, MRI is not limited by penetration depth, but on the other hand, it is not portable, expensive and has limited spatial resolution compared to HVM. In addition, MRI examinations are not suitable for critically ill patients in the emergency situation, but in the post-emergency situation, MRI can enable the assessment of organ anatomy, function, perfusion, oxygenation, alterations in the tissue texture and also metabolism. In this context, even mitochondrial function has been monitored by ^31^P NMR-spectroscopy in skeletal muscle mostly in sports-medicine (209). Moreover, the technical capabilities of MRI systems are rapidly expanding with higher field strength, the development and implementation of multichannel array coils, novel imaging techniques and innovative data acquisition schemes like compressed sensing that shorten imaging times and increase sensitivity. Another field with high developmental potential is MR-based molecular imaging. Detection of macrophages in liver tumors has been conducted in the past with paramagnetic iron oxide nanoparticles (210). The translational potential of ^19^F MRI is shown by the fact that PFCs have already been used in clinical trials as blood substitutes, that conventional ^19^F-based inflammation imaging is feasible with clinical MRI systems at 3T in large animals (211, 212) and also a cell tracking study using autologous DCs in human patients with colorectal cancer has been performed (213). ^19^F MRI is of particular interest, because there is negligible endogenous ^19^F background (214), the accumulation of ^19^F-atoms results in a true positive contrast that can be quantified and does not impair anatomical, functional and parametric ^1^H MRI. Additionally, ^19^F MRI is very versatile and multiple interesting approaches just recently emerged in the preclinical field like multispectral ^19^F MRI, environmental responsive smart probes or active targeting of ^19^F tracers (215, 216).

Finally, combining the information obtained by multiple types of devices for local microcirculation, oxygenation, function of mitochondria and organs with mechanistic insights derived from animal models could strongly support clinical decision-making and revolutionize therapy control in critical care medicine. Microcirculatory and mitochondrial monitoring could adjust global therapeutic approaches to the individual metabolic needs enabling differentiated vasopressor, fluid and transfusion regimes in the early course of disease. Later on, MR could identify tissues at risk as a consequence of temporary hypoxia, anaerobic metabolism and systemic and local inflammation. Paying attention to those critically compensated tissues and initiating organ-specific protective measures could avoid the entry in destructive cascades through an intensive organ cross talk and impede the development of multi organ failure.

## Author Contributions

SH, CM, AH, JS, RT, UF, and ST wrote individual chapters of the manuscript. IB, AR, OP, AK, SH, CM, JS, RT, UF, AH, and ST critically revised the whole manuscript. All authors contributed to the article and approved the submitted version.

## Funding

This work was supported by the Deutsche Forschungsgemeinschaft (UF: SFB 1116, TRR 259, FL303/6-1/2, INST 208/764-1 FUGG and ST: TE1209/1-1/2) and the European Commission (MSCA-ITN-2019 NOVA-MRI, MSCA-RISE-2019 PRISAR2 to UF).

## Conflict of Interest

The authors declare that the research was conducted in the absence of any commercial or financial relationships that could be construed as a potential conflict of interest.

## Publisher's Note

All claims expressed in this article are solely those of the authors and do not necessarily represent those of their affiliated organizations, or those of the publisher, the editors and the reviewers. Any product that may be evaluated in this article, or claim that may be made by its manufacturer, is not guaranteed or endorsed by the publisher.
