# Supplementary material for: A Toolbox to Investigate the Impact of Impaired Oxygen Delivery in Experimental Disease Models
Source: Front Med (Lausanne). 2022 May 16;9:869372. doi: 10.3389/fmed.2022.869372 (PMC9149176; doi:10.3389/fmed.2022.869372)
Supplement: Supplementary file 1 [file Table_1.pdf]

Supplementary Material

Supplementary Table S1: An extract of experimental preclinical animal studies of different shock-forms where imaging technologies have been used to detect structural and/or functional aspects of the microcirculation.

Author & Year	Shock-Form	Technology	Species	Brief summary	Ref.
Schulz *et al.* 2021	Sepsis	Spectro-photometry, video-microscopy	Rat	Subtherapeutic vasopressin application improves microvascular oxygenation and perfusion in a moderate model of sepsis. Therapeutic doses of vasopressin were not able to improve microvascular variables.	(1)
Truse *et al.* 2019	Hemorrhage	Spectrophotometry, video-microscopy	Dog	Topical losartan application improves gastric microcirculatory perfusion by local vasodilatation under otherwise physiological conditions. During mild hemorrhage losartan exerts minor effects on regional perfusion. Systemic macro-circulatory variables were not altered by topical pharmacological interventions.	(2)
Hessler *et al.* 2019	Hemorrhage and sepsis	Video-microscopy	Sheep	Total vessel density was barely affected by septic and hemorrhagic shock, whereas microvascular flow index and the perfused vessel density were impaired in septic and in hemorrhagic animals. Changes in conjunctival microcirculation were paralleled by changes in sublingual microcirculation.	(3)
Ferrara *et al.* 2017	Hemorrhage	Video-microscopy	Sheep	Intestinal and sublingual microcirculatory variables are impaired during HS. Both improved after blood transfusion. Thus, sublingual microcirculation might be adequate to track intestinal micro-circulation in a non-invasive manner.	(4)
Damiani *et al.* 2016	Sepsis	Video-microscopy	Rat	Microcirculatory variables are impaired in septic animals. The application of human serum albumin improved perfused vessel density, microvascular blood flow and microvascular flow heterogeneity. Higher doses achieved a longer lasting effect.	(5)
Zafrani *et al.* 2016	Sepsis	Palladium-porphyrin phosphor-rescence	Rat	Fluid substitution improved mean arterial blood pressure and renal blood flow without any impact on microvascular oxygenation. Blood transfusion was able to reestablish macrocirculation, microcirculation and renal oxygenation.	(6)

van Iterson *et al.* 2012	Hemorrhage	Phosphori-metry	Pig	Microvascular oxygen pressure in non-vital (e.g. gut) and vital (e.g. heart) organs are closely related to macrocirculatory variables in the acute phase of hemorrhage. Blood flow redistribution from non-vital to vital organs might be a phenomenon of an ongoing hemorrhagic shock.	(7)
Dubin *et al.* 2009	Hemorrhage	Video-microscopy, tonometry	Sheep	Microvascular flow index measured at the sublingual, intestinal serosal and intestinal mucosal surface decreases progressively in a model of HS. Those alterations were paralleled by an increase of lactate levels and more sensitive than intramucosal-arterial carbon dioxide content.	(8)
Fang *et al.* 2006	Hemorrhage and sepsis	Video-microscopy	Rat	Microcirculatory changes are more pronounced in septic animals than in individuals undergoing HS. Hemodynamic incoherence is existent in septic animals, while microcirculation is at least in part paralleled by macrocirculatory variables in hemorrhagic animals.	(9)
Schwarte *et al.* 2005	Hemodilution	Spectrophot ometry, palladium-porphyrin phosphoresc ence	Sheep	Hemodilution impairs intestinal oxygenation. Besides the redistribution of red blood cells as the main determinant of oxygen carrying capacity, microvascular shunting plays an important role in the development of microvascular failure.	(10)
Ferrara *et al.* 1985	Hemorrhage and hemodilution	Video-microscopy, tonometry	Sheep	The proportion of perfused vessels, the microvascular flow index and the red blood cell velocity decreased in hemorrhagic animals and under hemodilution. Changes were more pronounced in individuals receiving hemodilution.	(11)
Weidensteiner *et. al.* 2018	Septic shock	MRI	Mouse	Aim of the study was to monitor kidney function in septic animals (CLP) based on the accumulation and release of a gadolinium-based CA. After CLP, renal clearance of the CA was reduced as indicated by a slower wash-out from the renal cortex and medulla.	(12)
Towner *et al.* 2013	Septic shock	MRI	Mouse	*In vivo* detection of free radicals in murine septic encephalopathy after CLP. DMPO was used as a scavenger of free radicals that leads to the formation of DMPO-adducts. Administration of a gadolinium-labelled anti-DMPO probe, revealed accumulation of DMPO-adducts in septic animals by a change in T1 values in regions of the brain.	(13)
Foley *et al.* 2013	Hemorrhagic shock	MRI	Mouse	MRI using arterial spin-labelling was utilized to study the impact of HS in combination with traumatic brain injury on CBF. HS aggravated brain-injury induced reduction in CBF 90 min post HS. Of note,	(14)

				the impact on CBF showed variations depending on the region of the brain.	
Matot *et. al.* 2008	Hemorrhagic shock	MRI	Rat	T2*-weighted MRI of the liver after -HS and mapping of the relative changes in MRI-signal intensity which revealed decreased liver perfusion after HS.	(15)
Rosengarten *et al.* 2008	Septic shock	MRI	Rat	The purpose of this study was to detect sepsis associated brain edema by measurement of diffusion coefficients and T2-relaxation times by MRI. LPS was intravenously injected into male rats, but MRI revealed no alterations in signal intensities between control and septic animals in several regions of the brain.	(16)
Barash *et al.* 2007	Hemorrhagic shock	MRI	Rat	T2*-weighted MRI of the liver was performed to monitor perfusion and hemodynamics during hypercapnia and hyperoxia. Changes in signal intensity in the liver induced by hypercapnia and hyperoxia reflect changes in blood volume.	(17)
Maier *et al.* 2006	Hemorrhagic shock	MRI	Rat	Assessment of hepatocyte function and macrophage activity by contrast enhanced MRI after HS. Application of the liver-specific CA (Gd-EOB-DTPA) revealed lower hepatocyte activity after HS, whereas injection of superparamagnetic iron oxide nanoparticles did not show enhanced phagocytic activity of liver macrophages.	(18)
Dear *et al.* 2005	Sepsis	MRI	Mouse	MRI of acute kidney failure in aged mice after CLP or other forms of kidney injury (e.g. cisplatin or ischemia/reperfusion) using a gadolinium-based dendrimer. Dendrimer-enhanced MRI could distinguish CLP induced renal failure from other forms as early as 6 h post CLP, a time point where serum creatinine was not elevated.	(19)
Fuji *et al.* 1999	Septic shock	MRI	Rat	*In vivo* MRI detection of NO, which was complexed by (MGD)2-Fe(II) and imaged by EPR. The (MGD)2-Fe(II)-NO complex accumulated in the liver in those animals that were treated with intraperitoneal injection of LPS.	(20)
Li *et al.* 1998	Hemorrhagic shock	MRI	Dog	*In vivo* MRI of oxygenated hemoglobin in the superior mesenteric veins can be used to diagnose and monitor mesenteric ischemia.	(21)

Abbreviations: contrast agent (CA), cerebral blood flow (CBF), cecal ligation and puncture (CLP), 5,5-dimethyl pyrroline N-oxide (DMPO), electron paramagnetic resonance (EPR), Gadolinium-Ethoxybenzyl-Diethylenetriamin-Pentaacetic acid (Gd-EOB-DTPA hemorrhagic shock (HS), lipopolysaccharide (LPS), N-methyl-D-glucamine (MGD)2, iron in its oxidation state +2 (Fe(II)), magnetic resonance imaging (MRI), nitric oxide (NO).

References for supplementary table S1:

1. Schulz J, Bauer I, Herminghaus A, Picker O, Truse R, Vollmer C. Sub-therapeutic vasopressin but not therapeutic vasopressin improves gastrointestinal microcirculation in septic rats: A randomized, placebo-controlled, blinded trial. PLOS ONE. 2021;16(9):e0257034.

2. Truse R, Voß F, Herminghaus A, Schulz J, Weber APM, Mettler-Altmann T, et al. Local gastric RAAS inhibition improves gastric microvascular perfusion in dogs. J Endocrinol. 2019;241(3):235–47.

3. Hessler M, Arnemann P-H, Zamit F, Seidel L, Kampmeier T-G, Kathöfer U, et al. Monitoring of Conjunctival Microcirculation Reflects Sublingual Microcirculation in Ovine Septic and Hemorrhagic Shock. Shock. 2019;51(4):479–86.

4. Ferrara G, Edul VSK, Canales HS, Martins E, Canullán C, Murias G, et al. Systemic and microcirculatory effects of blood transfusion in experimental hemorrhagic shock. Intensive Care Med Exp. 2017;5(1):24.

5. Damiani E, Ince C, Orlando F, Pierpaoli E, Cirioni O, Giacometti A, et al. Effects of the Infusion of 4% or 20% Human Serum Albumin on the Skeletal Muscle Microcirculation in Endotoxemic Rats. PLOS ONE. 2016;11(3):e0151005.

6. Zafrani L, Ergin B, Kapucu A, Ince C. Blood transfusion improves renal oxygenation and renal function in sepsis-induced acute kidney injury in rats. Crit Care. 2016;20(1):406.

7. van Iterson M, Bezemer R, Heger M, Siegemund M, Ince C. Microcirculation follows macrocirculation in heart and gut in the acute phase of hemorrhagic shock and isovolemic autologous whole blood resuscitation in pigs. Transfusion (Paris). 2012;52(7):1552–9.

8. Dubin A, Pozo MO, Ferrara G, Murias G, Martins E, Canullán C, et al. Systemic and microcirculatory responses to progressive hemorrhage. Intensive Care Med. 2009;35(3):556–64.

9. Fang X, Tang W, Sun S, Huang L, Chang Y-T, Castillo C, et al. Comparison of buccal microcirculation between septic and hemorrhagic shock. Crit Care Med. 2006;34(12 Suppl):S447-453.

10. Schwarte LA, Fournell A, van Bommel J, Ince C. Redistribution of intestinal microcirculatory oxygenation during acute hemodilution in pigs. J Appl Physiol. 2005;98(3):1070–5.

11. Ferrara G, Kanoore Edul VS, Martins E, Canales HS, Canullán C, Murias G, et al. Intestinal and sublingual microcirculation are more severely compromised in hemodilution than in hemorrhage. J Appl Physiol. 15. Mai 2016;120(10):1132–40.

12. Weidensteiner C. Reichardt W., Struck J., Kirchherr A., Wagner K., Wagner F., von Elverfeldt D. Analysis of kidney function and therapy monitoring with DCE-MRI in a murine model of septic shock. 2018. Proc. of ISMRM 2018; https://index.mirasmart.com/ISMRM2018/PDFfiles/4599.html

13. Towner RA, Garteiser P, Bozza F, Smith N, Saunders D, d'Avila JCP, et al. In vivo detection of free radicals in mouse septic encephalopathy using molecular MRI and immuno-spin trapping. Free Radic Biol Med. 2013;65:828–37.

14. Foley LM, O'Meara AMI, Wisniewski SR, Hitchens TK, Melick JA, Ho C, et al. MRI Assessment of Cerebral Blood Flow after Experimental Traumatic Brain Injury Combined with Hemorrhagic Shock in Mice. J Cereb Blood Flow Metab. 2013;33(1):129–36.

15. Matot I, Cohen K, Pappo O, Barash H, Abramovitch R. Liver response to hemorrhagic shock and subsequent resuscitation: MRI analysis. Shock. 2008;29(1):16–24.

16. Rosengarten B, Walberer M, Allendoerfer J, Mueller C, Schwarz N, Bachmann G, et al. LPS-induced endotoxic shock does not cause early brain edema formation – An MRI study in rats. Inflamm Res. 2008;57(10):479–83.

17. Barash H, Gross E, Matot I, Edrei Y, Tsarfaty G, Spira G, et al. Functional MR Imaging during Hypercapnia and Hyperoxia: Noninvasive Tool for Monitoring Changes in Liver Perfusion and Hemodynamics in a Rat Model. Radiology. 2007;243(3):727–35.

18. Maier M, Hahn P, Schneider G, Marzi I. Magnetic Resonance Imaging (MRI) for Non-invasive Analysis of Hepatic Function After Hemorrhagic Shock in the Rat. Eur J Trauma. 2006;32(5):449–55.

19. Dear JW, Kobayashi H, Jo S-K, Holly MK, Hu X, Yuen PST, et al. Dendrimer-enhanced MRI as a diagnostic and prognostic biomarker of sepsis-induced acute renal failure in aged mice. Kidney Int. 2005;67(6):2159–67.

20. Fujii H, Wan X, Zhong J, Berliner LJ, Yoshikawa K. In vivo imaging of spin-trapped nitric oxide in rats with septic shock: MRI spin trapping. Magn Reson Med. 1999;42(2):235–9.

21. Li KC, Pelc LR, Puvvala S, Wright GA. Mesenteric ischemia due to hemorrhagic shock: MR imaging diagnosis and monitoring in a canine model. Radiology. 1998;206(1):219–25.
